# The use of spreadsheets with analytical instrumentation for laboratory automation

**DOI:** 10.1155/S1463924690000190

**Published:** 1990

**Authors:** John M. Graft

**Affiliations:** National Instruments 6504 Bridge Point Parkway Austin Texas 78730 USA


Journal of Automatic Chemistry, Vol. 12, No. 4 (July–August 1990), pp. 149–152

### The use of spreadsheets with analytical instrumentation for laboratory automation

**John M. Graff**

*National Instruments, 6504 Bridge Point Parkway, Austin, Texas 78730, USA*

### Introduction

Scientists today are finding that they cannot adequately perform their jobs using manual experimentation methods. The costs, inefficiencies and potential for errors are simply too high. The low cost and increasingly high performance of personal computers has led many scientists to choose such machines as the foundation for automating their laboratories. In addition, a personal computer provides a wealth of software to perform analysis, databasing, graphics, word-processing, and many other functions. Performing a procedure, presenting results, or maintaining archival data are standard day-to-day tasks for a scientist.

### Instruments and the personal computer in the laboratory

The first step toward automation in many laboratories is the use of a computer to analyse and store experimental data. The methods for performing these tasks range from user-written BASIC programs and general-purpose spreadsheet packages to specialized analysis packages. Although the spreadsheet is best known for its business applications, it can safely be called the most popular analysis software ever written for a PC. A spreadsheet is a natural place to tabulate, analyse, and display data, as many scientists have discovered. The problem for many scientists who have used a spreadsheet for analysis and presentation of data is that they could do so only by manually entering the data or storing it in an ASCII file and importing it into a spreadsheet.

At the same time that the personal computer has made computing power more accessible, instruments have been designed to be faster and more sensitive. Instruments can often transfer their data directly to a personal computer. The RS-232 serial interface is often the standard interface (or at least an optional interface) for most types of instruments found in the laboratory, while the IEEE-488 (also called GPIB or HP-IB) interface is increasingly found on analytical instruments. These communications protocols are used to transfer an instrument's data to a personal computer for analysis, graphic display, and storage (figure 1).

However, the interface on the instrument is only the first step. The software running on the personal computer

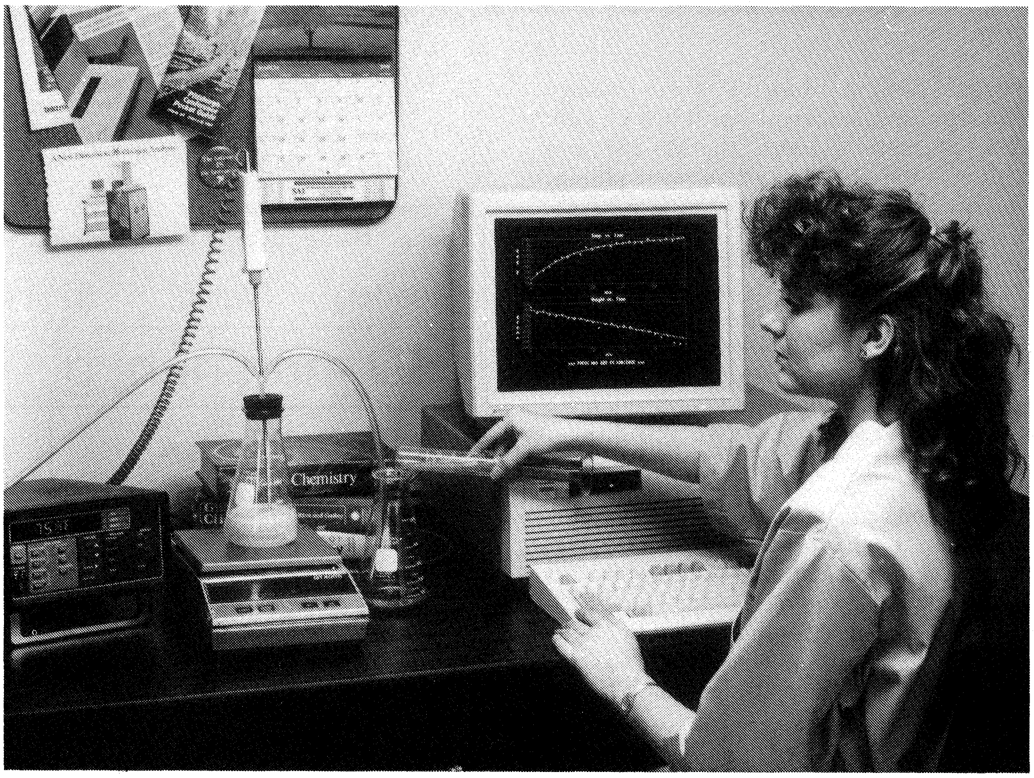

*Figure 1. Personal computers are becoming the foundation for laboratory automation.*

0142–0453/90 $3.00 © 1990 Taylor & Francis Ltd.

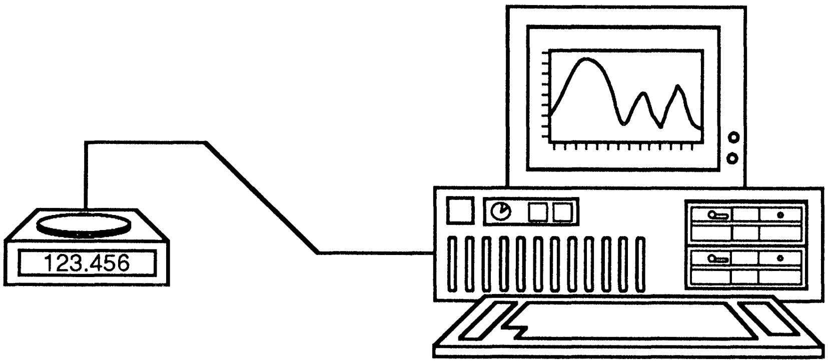

*Figure 2. Example set-up of personal computer and laboratory balance.*

must be flexible enough to accept data under a variety of conditions and in a number of different formats. The personal computer must be flexible enough to accommodate an instrument's fixed data transfer settings, such as baud rate, parity, and data length.

Most instruments send data as a string of ASCII alphanumeric characters, often sending several pieces of information in a single transmission. As a result, the data received by the computer must be separated or parsed into meaningful groupings, or elements, of data before they are usable. These include header information, numbers, words and status indicators. Parsing can be a complicated task, since data formats vary greatly between instruments and the length of a data element may vary, both between different elements in a single transmission and in the same element in sequential transmissions. This paper focuses on the use of the Lotus 1-2-3 and Measure software packages. This software combination forms a comprehensive data acquisition and analysis package that meets a range of general requirements for laboratory automation.

### Measure description

Measure is a personal computer-based software package that collects data from measurement hardware directly into Lotus 1-2-3. Measure supports the RS-232 and IEEE-488 communications buses, and selected plug-in data acquisition boards.

Measure is fully integrated with 1-2-3, creating a single, consistent software environment. Measure shares the menu-oriented user interface of 1-2-3, so that it will be familiar to those who work with 1-2-3. In addition, Measure shares the 1-2-3 macro environment. A single macro can incorporate both Measure and 1-2-3 functions to perform an entire collection, analysis, graphic display and storage procedure.

Measure places acquired data directly in a 1-2-3 worksheet, without having to create an intermediary file. Measure supports full bidirectional communications with instruments through RS-232 and IEEE-488 interfaces; not only is incoming data stored immediately in worksheet cells, but data or commands in worksheets cells can be sent over the bus to the remote instrument for setup or control.

An important aspect of Measure is its ability to handle instrument data. Handling data means formatting the data so that it is immediately usable in the worksheet. In the RS-232 environment, the major challenge of handling data is in parsing the data into a useful form.

### Using Measure with a laboratory balance

Here is an example to illustrate how Measure facilitates the transfer and handling of data in 1-2-3. The hardware set-up consists of a Mettler PM4600 top-loading laboratory balance, with the optional RS-232 interface, and an IBM PC (figure 2). A standard RS-232 cable connects the balance to a serial port on the PC.

### *Parsing the incoming data*

The PM4600 is similar to many other RS-232 compatible instruments, because of its complex data format. Each data point, when transmitted across the bus, consists of three elements: (1) a header, either one or two characters long, indicating whether the reading is stable or dynamic; (2) the numerical value, which is of variable length; and (3) the unit of measure, which is invariably g for grams. One or more space characters separate the three data elements. Ideally, the three elements should be parsed upon receipt so that each group is stored in a separate cell in the worksheet, as shown in figure 3. This allows statistical analysis to be performed on the numerical data in column B. The label information in column A can be used to sort the data by the type of reading, using the 1-2-3 database functions.

Measure has two methods available to accomplish this parsing. Using the first method, data is separated

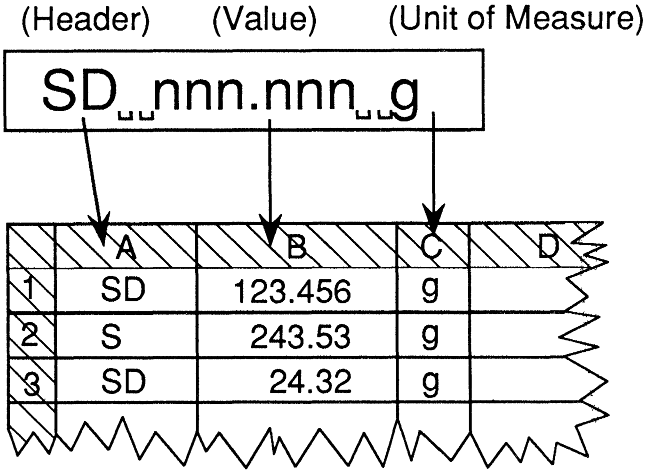

*Figure 3. Data format of the Mettler PM4600 balance. When parsed on the space character, data is stored in 1-2-3 as shown.*

according to character count, so that the first $x$ characters are stored in the first cell, the next $y$ characters are stored in the next cell, and so on. This method is appropriate for the fixed-length data elements used by some instruments.

However, as noted earlier, the balance's data elements are of variable length. A second method is available. Measure uses a specified character in the transmitted string to indicate when one element ends and the next begins. This character is called the *element separator.* For the data from the balance, the space character (ASCII 32) is the appropriate element separator. When Measure parses on this character, the result is as shown in figure 3. Note also that the first and third elements, which consist of alphabetic characters, are stored as labels. The second element, which is a number, is automatically stored as a number. It can be included in mathematical calculations without any further manipulation.

*Stripping unwanted data elements*

As instruments become more sophisticated internally, output formats often reflect that increased complexity. Instruments often add cryptic headers to the front of data, while status information used only by the instrument is appended to the end. To the scientist who wants the data, this additional information hides the data and uses up valuable storage space.

Measure allows the user to strip off unwanted elements from data as it is received. After the data is parsed into separate elements using one of the methods described above, Measure can selectively store the elements. This selection is specified using an *include table* that is designated in Measure.

In the example of the laboratory balance, a user may not need to know whether the weight at measurement reading is stable or changing, or that every reading is in grams, since they all are. Therefore, the first and third of the three data elements may be stripped off, leaving only the weight. The user specifies this from the Measure menu; the resulting data collection is shown in figure 4. This solution saves storage space, permits better worksheet organization, and stores data in the most useful format immediately.

### Using Measure for automated titration

Titration is a quantitative methodology frequently used in analytical chemistry. The tedium of measuring out reagent and checking for a reaction has been relieved by the combination of hardware and the Measure software. Modern titrators can automatically titrate a sample, while measuring the electrode values. Some can be combined with sample changers to titrate 20 or more samples without human intervention. Because most of this equipment supports RS-232, Measure can be used to set up and control the titration station, placing the data directly in 1-2-3 for display, analysis, and storage.

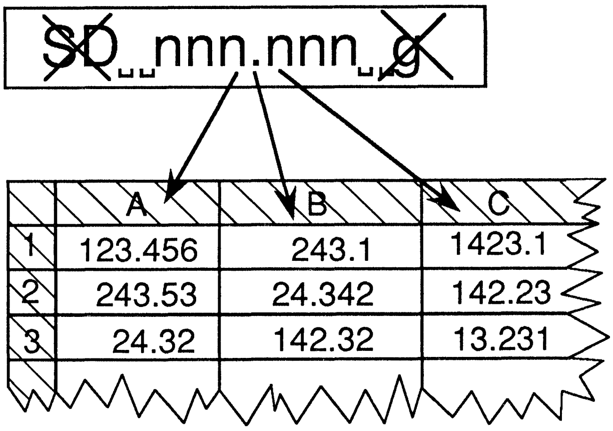

*Figure 4. Storage of data from the balance, using a space character as delimiter and an* include table *to strip off the first and third data elements.*

*Set-up*

The set-up is depicted in figure 5, using a Mettler DL20 Compact Titrator and ST20 Sample Changer to actually perform the titration. The combination of these instruments can automatically titrate up to 20 100 ml samples without the need for human intervention. A bar-code scanner is used to identify the samples when they are put in the sample changer, both to track the samples and also to determine the specific method of titration to use for each sample. To connect the three instruments, all RS-232 compatible, to the personal computer, a Source For Automation PC-232 Intelligent Port Expander is used, providing eight RS-232 ports while requiring only one port on the PC.

*Procedure*

A macro running on the PC controls the procedure. Running under 1-2-3, and incorporating 1-2-3 and Measure functions, it provides a menu environment that allows the user to choose the next action. The macro itself creates and transmits the ASCII character strings to set up and control the titrator and sample changer, while also taking in data from the titrator and bar-code scanner.

At the beginning of the procedure, the bar codes are scanned from all of the samples and stored in the 1-2-3 worksheet. As each sample is then placed by the sample changer under the titration head, that bar-code information is used to choose the titration method for that sample. As a result, different types of samples may be run in the same batch, assuming of course that they are using the same titrant in their methods. At the completion of each titration, the data is requested from the titrator; it too is stored in the worksheet. The data can then be graphed automatically in 1-2-3 with either the raw data (figure 6) or analysed data, such as the slope (figure 7).

### Conclusion

A personal computer can be a powerful and versatile foundation for automating a laboratory. The move to a personal computer for this purpose obviously requires

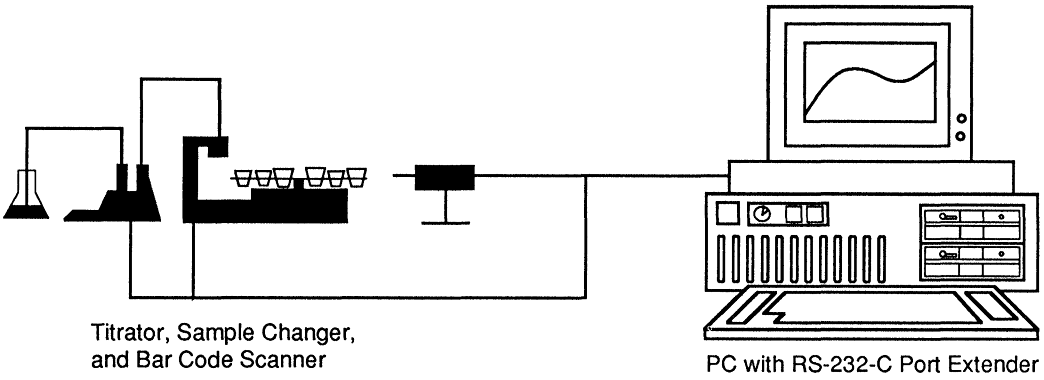

*Figure 5. Hardware set-up for automated titration.*

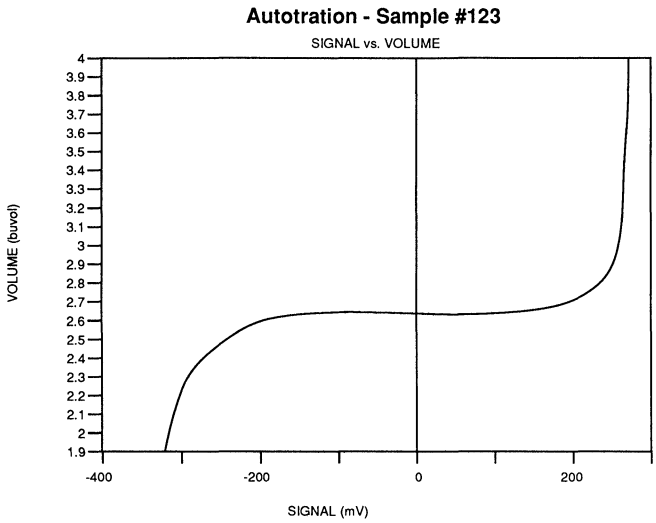

*Figure 6. Typical titration curve of volume versus signal.*

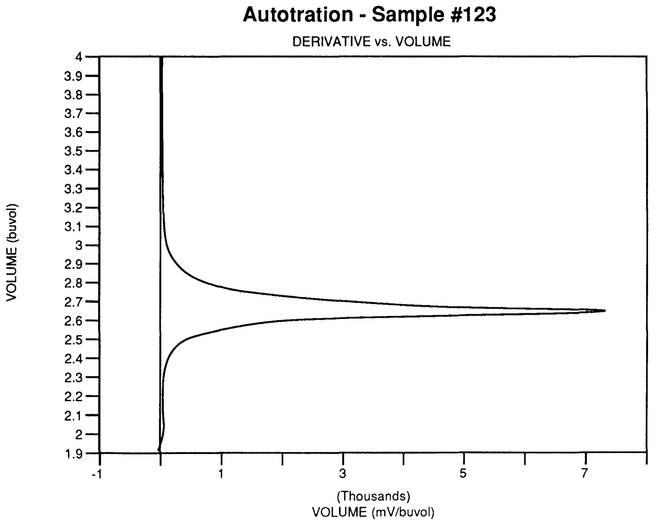

*Figure 7. Typical titration curve of volume versus first derivative.*

many considerations beyond the choice of a computer with the right hardware options. Much thought must be given to the means and methods used to import the data into the selected computer. The important aspect to keep in mind is that the entire process will progress more smoothly if the various phases of the application, from acquisition to analysis to presentation, can be performed in a common and consistent manner. In particular, the more seamless the integration of the acquisition process with post acquisition tasks, the more efficient and straightforward will be the job of getting the application up and running.

